# From Raising Awareness to a Behavioural Change: A Case Study of Indoor Air Quality Improvement Using IoT and COM-B Model

**DOI:** 10.3390/s23073613

**Published:** 2023-03-30

**Authors:** Rameez Raja Kureshi, Dhavalkumar Thakker, Bhupesh Kumar Mishra, Jo Barnes

**Affiliations:** 1 School of Computer Science, University of Hull, Kingston upon Hull HU6 7RX, UK; r.kureshi@hull.ac.uk (R.R.K.); bhupesh.mishra@hull.ac.uk (B.K.M.); 2 Air Quality Management Resource Centre, University of the West of England, Bristol BS16 1QY, UK; jo.barnes@uwe.ac.uk

**Keywords:** indoor air quality (IAQ), raising awareness, indoor activities, Internet of Things (IoT), behaviour science, behaviour change

## Abstract

The topic of indoor air pollution has yet to receive the same level of attention as ambient pollution. We spend considerable time indoors, and poorer indoor air quality affects most of us, particularly people with respiratory and other health conditions. There is a pressing need for methodological case studies focusing on informing households about the causes and harms of indoor air pollution and supporting changes in behaviour around different indoor activities that cause it. The use of indoor air quality (IAQ) sensor data to support behaviour change is the focus of our research in this paper. We have conducted two studies—first, to evaluate the effectiveness of the IAQ data visualisation as a trigger for the natural reflection capability of human beings to raise awareness. This study was performed without the scaffolding of a formal behaviour change model. In the second study, we showcase how a behaviour psychology model, COM-B (Capability, Opportunity, and Motivation-Behaviour), can be operationalised as a means of digital intervention to support behaviour change. We have developed four digital interventions manifested through a digital platform. We have demonstrated that it is possible to change behaviour concerning indoor activities using the COM-B model. We have also observed a measurable change in indoor air quality. In addition, qualitative analysis has shown that the awareness level among occupants has improved due to our approach of utilising IoT sensor data with COM-B-based digital interventions.

## 1. Introduction

Breathing healthy air in all environments, including indoors and ambient (outdoors), is a fundamental human right [1]. Healthcare institutes, communities, and local authorities are concerned about air pollutant exposure, as air pollution plays a significant role in developing and exacerbating human respiratory issues [2]. Several air pollutants such as carbon dioxide (CO_2_), carbon monoxide (CO), particulate matter (PM_2.5_ and PM_10_), sulphur oxides (SO_x_), nitrogen dioxide (NO_2_), and some unburned hydrocarbons can create an unhealthy breathing environment. These pollutants can cause respiratory health issues such as asthma, they can affect the cardiovascular system, and in some cases, they might lead to cancer [3,4,5]. Air pollution can be indoor or outdoor; however, people still believe that outdoor air quality (OAQ) has more influence on human health than indoor air quality (IAQ). However, in reality, IAQ is 3–5 times worse than OAQ, as we spend more than 90% of our time in the indoor environment [6,7]. For this reason, the topic of indoor air quality has started to receive more attention in the recent decade.

Furthermore, IAQ directly affects human behaviour, performance, and productivity, especially for those who primarily work indoors [8]. Additionally, indoor activities such as cooking and cleaning and how frequently and long windows are opened during cooking influence indoor air quality. Human behaviour plays a significant role in affecting indoor air quality. Factors such as human attitudes and beliefs, socioeconomic status, and education level play a crucial role in shaping an individual’s behaviour towards air quality awareness [9]. Several studies [10,11,12,13,14] have been conducted using sensor technologies to monitor IAQ and raise citizens’ awareness. However, methodological approaches that utilise well-known behaviour models to influence behaviours with the help of sensor data visualisation are an open subject of exploration. In this paper, the following two Research Questions (RQ) are articulated to investigate air quality awareness and the impact of behavioural interventions on raising awareness.

RQ1 (study 1): How to raise awareness about IAQ with the help of an IAQ monitoring sensor device and a daily digital diary?

RQ2 (study 2): How to influence and evaluate citizens’ behavioural changes concerning IAQ by operationalising a behavioural psychology theory?

The first pilot case study was conducted with ten households in Bradford with volunteer participants. This study aimed to raise awareness regarding the IAQ with the help of real-time IAQ data and by filling a daily digital diary to understand the context of indoor activities. From analyses of this study data, it has been observed that there has been an appreciable increase in citizens’ awareness towards IAQ. Despite the observed behavioural change, no conclusive evidence exists on which factor influences raising the citizens’ awareness. The second case study was conducted with eight different participants in the next stage to address this aspect. The COM-B model was utilised in study 2 to design digital interventions and measure behavioural change using Internet of Things (IoT) technology. The analytical results from study 1 have shown that there has been an increase in window opening hours ranging from 11% to 39%, reflecting self-awareness towards IAQ. In study 2, the digital interventions significantly changed the participants’ behaviour, impacted indoor activities, and improved overall IAQ. As far as we know, this is the first work of its kind to use a behaviour psychology model, COM-B, to design and operationalise digital interventions as part of a digital tool in terms of bringing about behaviour change concerning IAQ. 

The rest of the paper is organised as follows: Section 2 comprehensively reviews IAQ, its health impact, and behavioural change studies. Then, in Section 3, study 1 is presented with the study’s design, data analysis, experimental results, and conclusion. In Section 4, study 2 is presented with the design, data interpretation, experimental results, and analysis of the study. Finally, in Section 5, the paper is concluded with a discussion on future work.

## 2. Literature Review

This section presents a comprehensive review covering different aspects of IAQ, citizen awareness, and influential factors for behavioural change. First, the review highlights the IAQ in relation to human behavioural activities and health issues. It also covers how the digital platform has been used for behavioural change analysis by applying different methodologies, including low-cost sensors (LCS). Finally, the review also focuses on the behavioural model to influence awareness with regard to air quality.

### 2.1. Health Issues in Congruence with IAQ and Human Behaviour and Activities

IAQ is mainly influenced by either human indoor activities, such as cooking and cleaning, building characteristics, or external parameters, such as ambient environmental conditions [15]. The Institute of Medicine, UK report has shown that human behaviour and the properties of pollutants are among the significant factors influencing the IAQ [16]. Human behavioural activities are directly associated with the emission of chemical components that react and increase indoor pollution levels according to their environmental characteristics. For example, in most cases, PM and COx are generated through cooking and combustion activities such as wood burning and smoking, which can lead to chest pain, aggravated asthma, fatigue, and decreased lung functionality if the exposure level is acute in the indoor environment [17]. Furthermore, acute concentrations of Total Volatile Organic Compounds (TVOCs), a group of organic substances emitted from paints, cleaners, and air fresheners, have an impact on human health, such as ear, nose, and throat (ENT) irritation, nausea, and headaches [18]. The pollution exposure level for humans depends not only upon their working schedule and outdoor weather conditions but also on their indoor behavioural activities such as daily chores. Moreover, citizens’ other indoor activities, such as opening and closing doors and windows and ventilation systems, also vary the indoor pollution level [4,19,20]. 

Over the years, researchers have presented different household components that directly or indirectly have a relationship with IAQ. Li et al. [21] argued that bioaerosol and ventilation systems, cleaning solutions, and detergents could cause an increment in indoor air pollution levels [22]. Additionally, a study conducted by Heo et al. [19] showed that the number of people present in a chamber is directly correlated with the concentration of bacterial bioaerosols (airborne biological particulate matter) and hence, the air pollution level [23]. Similar other studies [24,25,26] have shown that indoor occupants’ walking activity also influences aerosol particle concentration. Likewise, studies concerning IAQ [27,28] have shown that air particle filtration usage significantly impacts indoor air quality improvements, especially for houses with allergies or asthma patients. Tran et al. [29] mentioned the symptoms caused by poor IAQ associated with Sick building syndrome (SBS) and categorised them into four parts, namely, (i) neurotoxic effects: headaches, irritability, and fatigue, (ii) mucous membrane irritation: ENT irritation, (iii) gastrointestinal problems, skin irritation, and dryness, and (iv) asthma and asthma-like symptoms: chest tightness and wheezing, etc. They also discussed the health impacts of IAQ on the elderly, infants, and people suffering from chronic diseases. There are guidelines for improving human health concerning indoor environments. The World Health Organization (WHO, 2010) published guidelines to prevent public health risks associated with various air pollution exposure levels in the indoor environment [30]. Considering the IAQ, the National Institute for Health and Care Excellence (NICE), England, also issued guidelines in 2020 to raise citizens’ awareness to achieve good indoor air quality [31]. Despite having guidelines for different IAQ pollutants, their exposure levels and their control measures, the guidelines, and reference values are not cohesive, as they sometimes can sometimes be contradictory. [32]. 

Moreover, improving IAQ and raising awareness based on behavioural changes is now the centre of attraction for the research community and government bodies. However, the impact of this awareness is futile if the method of communication is ineffective for the targeted audience [33]. Lin et al. [20] investigated human behaviour and indoor air quality in a smart home environment, focusing on occupants’ indoor activities using ambient sensors that monitor motion, doors, light, and temperature. In addition, the device also measured air pollutants such as PM, O_3_, CH_4_, NO_x_, and CO_2_. This study concludes a strong relationship between in-home human activities and IAQ. The study has also focused on the indoor temperature to identify any indoor activity occurrence since indoor activities impact the temperature. It has been argued that the temperature remains unchanged for a longer period despite the indoor activity that has been performed earlier. Correspondingly, this can correlate the IAQ data with the indoor activity that happened during the same period of time. Some other studies [34,35,36,37] have focused on IAQ-related human behaviour, such as opening windows for good ventilation. On the other hand, some of the studies [38,39,40] have suggested that cooking activity has the most considerable impact on IAQ compared to any other indoor activities.

In addition, citizens’ income [41,42], house characteristics [43], and their social diversity [44] can also affect the IAQ. Brown et al. [45] conducted a study in France to find the relationship between socioeconomic status (SES) and IAQ. In their study, they observed that households with lower incomes have more likely to have high indoor pollution levels. A similar study led by Rumchev et al. [43] among women and children in some urban parts of India showed that lower incomes and indoor smoking significantly impacted their well-being. The social diversity of people in every country significantly affects IAQ due to the diversity in lifestyle and cultural practices [44,46]. An experimental study conducted by Walton et al. [47] in east London (UK) examined 333 children (8–9 years) of different ethnicities. This study found that prolonged air pollution exposure (PM and NOx) considerably impacted telomeres length, leading to ageing and immunological senescence in a later stage of life.

### 2.2. IAQ Monitoring Studies Using Low-Cost Sensors (LCS): A Citizen Engagement Approach

Considering the IAQ and the amount of time citizens spend indoors, further studies are required on IAQ to establish links between citizens’ indoor activities and IAQ. For sustainable smart city solutions, one of the major concerns is reducing air pollution and raising awareness about it at the citizen’s level. To achieve this, researchers have implemented different infrastructures that can monitor air quality with the IoT framework, both outdoors [12,13] and indoors [11,14]. Such air quality (AQ) monitoring infrastructure can raise AQ awareness and build sustainable smart city solutions [48,49]. However, conventional AQ monitoring systems have substantial challenges, such as the high cost of devices, often ranging in tens of thousands of US dollars, their coverage area, and their large size [7]. Because of these challenges, the existing air quality monitoring stations have a lower area coverage as it is not feasible to deploy such devices at a large scale. As an alternative, LCS have revolutionised the approach in this domain for the last few years as these LCS devices have lower costs and are compact and portable, making these devices viable. In addition, these LCS devices provide real-time and high-resolution spatiotemporal data to the operator at any specific location [50]. Therefore, to monitor IAQ, LCS provide an economical solution compared with more expensive sensor systems such as regulatory monitors. However, LCS have challenges in terms of data accuracy that are being addressed in LCS device design using approaches such as calibration [51,52]. 

Cities with an infrastructure that can monitor air quality by employing LCS devices can help the government, local authorities, and citizens keep track of air quality compared with high-cost sensor devices. These LCS devices also raise citizens’ awareness and help them understand AQ in a better way. For example, Willet et al. [53] used their personal AQ monitoring sensors and interviews to design a framework and principles for data collection. Zappi et al. [54] studied the responses of the citizens and their understanding of the air quality in the surroundings. Castell et al. [55] provided LCS-based mobile devices to the smart city, which monitor AQ and ease their participation in governing environmental air quality. Jarret et al. [56] showed that an LCS-based monitoring system had the potential to obtain data as a valid data source to evaluate citizen science studies further. Hubbell et al. [57] concentrated on a comprehensive study of people’s approaches, behaviour, and opinion related to AQ sensor use. The author also scrutinised the collaborative approach between citizen scientists and citizens that resulted in improved sensor technology as well as contributed to raising AQ awareness.

These studies have argued that citizens’ indoor activities influence indoor pollution levels and human health. Unmonitored indoor activities and a lack of citizens’ awareness of indoor pollution levels can further increase human health risks. Considering this, an indoor pollution monitoring system with a log of indoor activities can play a vital role in improving citizens’ indoor air pollution awareness and influencing behaviour change. Real-time IAQ data availability can impact their behaviour change, increasing their engagement to curtail indoor air pollution levels and influential indoor activities. IoT-enabled LCS devices and interactive IoT platforms can be a practical alternative to achieve this at the citizens’ household level. 

### 2.3. Changes in Human Behaviour through Ventilation for Raising Awareness Regarding IAQ

Several studies have shown that it is crucial to raise awareness about IAQ among citizens [3,37]. Moore et al. [10] have shown that measuring and presenting air quality readings to the citizens raised awareness. Ventilation is essential to maintaining good AQ in the indoor environment. Ventilation removes contaminants (dust and humidity) and recycles fresh air for breathing. In the scenarios when sufficient fresh air is not flowing into the room by existing ventilation systems or when the quality of this air is poor, problems arise, especially with the health risks, including respiratory infections and aggravation of allergies. Achieving an appropriate level of indoor comfort depends on several factors that relate to human behaviour and the design of architectural spaces: precisely, ventilation rate, thermal comfort, lighting control, house layout, and reorganisation. Experts have suggested many techniques to enhance IAQ and lower indoor air pollution levels, including air ventilation [58,59]. IAQ improvement and awareness-raising based on behavioural changes are currently the research community’s and governmental organisations’ focus [33]. However, if the chosen communication strategy is ineffective for the intended audience, the influence of this awareness is useless. That is why it is essential to pay attention to the quality of air that citizens inhale at home in order to bring good health and well-being across their lifespan.

### 2.4. Digital Health and Behavioural Change

Citizen engagement towards health is a psychosocial progression resulting from the behavioural representation of individuals. Support from citizens in digital health interventions can improve their engagement towards their health condition [60]. Considering the digital platform enhancement, data-driven analytical approaches increase the effectiveness of a range of behavioural health outcomes. Improved digital technology has given the opportunity for behavioural health interventions, health messaging, and accessing specific data. The digital platform also provides the opportunity to increase the effective influence of behavioural change towards health [61]. Besides this, a digital platform is a scalable tool that has significant potential to improve personalised health awareness and raise consciousness through digital health intervention [62,63,64]. 

Air pollution has been linked to a number of health issues. Different forms of interventions can be used to improve the citizens’ health status. For example, individual-level interventions have been implemented to minimise exposure levels to air pollution among citizens with long-term respiratory conditions [65]. The study results have shown that there have been improvements in the intervention group, whereas another group showed only minor improvements in pollution exposure levels. In another study on air quality and its health impact, personalised data and public engagement have been used to support citizen action towards minimising the health issues from air pollution [63]. The study by Sater et al. [66] applied generic and personalised interventions to raise indoor air pollution awareness at household levels. The study results show that only personalised intervention raised the awareness level of the intervention group compared to the control group. Apart from personalised intervention, community counselling has also been applied to children below five years of exposure to PM_10_ and CO levels using a quasi-experimental design. The study involved intervention and control communities revealing that indoor air pollution was reduced in both communities. However, the intervention group performed better than the control group [67]. Mouri et al. [68] applied physical interventions to explore the impact of exercise among elderly citizens to explore if there is any association between changes in quality of life and behavioural change. The results showed that there were substantial differences in quality of life between the citizens who follow the exercise schedule and those who did not. Fan et al. [69] conducted a field study of the effects of the bedroom window and door opening hours concerning IAQ, sleep quality, and next-day cognitive performance. The analytical result showed that interventions in opening windows and doors are required to achieve good IAQ, sleep quality, and individual human behaviour.

## 3. Design of Study 1: To Increase Awareness of Indoor Air Pollution with IoT 

This study aimed to trial the use of an IoT device for IAQ monitoring and to evaluate if showing a visualisation of IAQ data leads to increased awareness among participants. The study plan went through an ethical approval panel from the University and was approved by the Chair of the Biomedical, Natural, Physical, and Health Sciences Research Ethics Panel.

### 3.1. Study Instrument and Tools: IoT Device for IAQ, Visualisation Platform with Daily Digital Diary

We designed LCS-based IoT devices for reliable IAQ monitoring in our previous work. This device has been built with different low-cost sensors, as shown in Table 1, with the capability of monitoring air pollutants such as PM (PM_2.5_ and PM_10_) and meteorological parameters such as temperature and humidity. This device is calibrated for PM_2.5_ and PM_10_ with high-fidelity reference air quality monitoring stations using Machine Learning (ML) techniques [70,71]. We approach four calibration algorithms: MLR (multiple linear regression, MLP (multi-layer perceptron), CNN (convolutional neural network), and RF (random forest) to find out the best-suited calibration model for the selected sensor. The RF algorithm appeared to be the best model for calibrating LCS among these four algorithms. These calibrated devices need to get connected to the main power supply, and they need Wi-Fi for transmitting data. The device collects and sends the IAQ data every 15 min to the cloud server for processing. 

**Figure 1:**
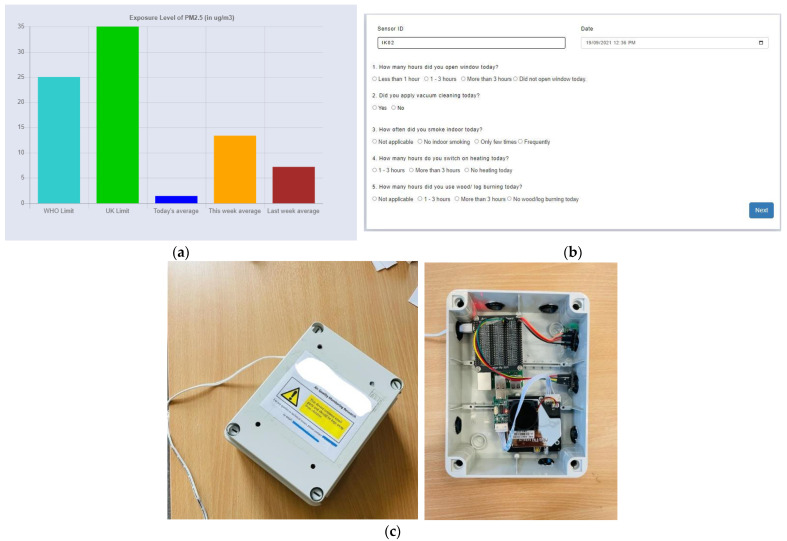
(**a**) IAQ data comparing with UK and WHO limits. (**b**) Digital daily activity log form. (**c**) LCS-based IoT assembled kit deployed at participant’s house.

In addition, an interactive digital platform has also been designed for IAQ data visualisation and capturing daily indoor activities. The visualisation shows PM_2.5_ and PM_10_ data with five different plots: WHO limit, UK limit, today’s average value, this week’s average value, and last week’s average value. These five different plots allow participants to compare their indoor air pollution readings with two defined guidelines from the WHO and the UK. The daily digital diary contains nine interactive multiple-choice questions in three steps: opening windows, vacuum cleaning, breathing problems, smoking, heating, and cooking, as shown in Figure 1. These questions are designed based on the literature related to socio-diversity and air quality-related health impacts [72,73,74]. This study went through a health and safety approval focusing on COVID-19-related adjustments.

### 3.2. Study Location, Participants, and Study Context

The study was a part of two European Union-funded projects, SCORE (https://northsearegion.eu/score/ (accessed 7 March 2021)) and LifeCritical (https://lifecritical.eu/ (accessed on 12 April 2021)). The SCORE project focuses on building smart city solutions with citizen engagement. The LifeCritical project focuses on utilising parks and green spaces for climate adaptation and resilience. The study participants were chosen from the Horton Park area in Bradford, a city in the north of the United Kingdom. The Bradford Metropolitan District Council (BMDC), a partner in the two projects, played a crucial role in connecting the researchers with the community group and establishing the initial contact points. The participants were chosen through a community group called Friends of Horton Park, which is an active community group living in and around Horton Park. The BMDC statistics show that 8.9% of homes in the Horton Park area are overcrowded, which is higher than the district average. The life expectancy for men living in this area is lower than the district average. A total of 44.4% of homes in the Horton Park area are terraced, 38.3% are semi-detached, 9% are detached, and 8.3% are flats. These houses were generally built at low cost and, if not adequately maintained and modernised, they will be found to have problems such as dampness, poor insulation, cracks in walls, and roofs in need of repair.

The study was conducted between September and October 2021 for two months in Bradford City for eight weeks. Altogether, ten households were selected based on socioeconomic and demographic variables such as location, ethnicity, and house type. The household had a designated person responsible for accessing IAQ data, disseminating it to the household, and filling in the daily digital diary for the household. The assembled IAQ monitoring kit, as shown in Figure 1c, has been deployed at participants’ houses. After the device deployment process, the research team did a session with each household on using the IAQ visualisation platform and filling the daily digital diary. For the participant’s privacy, there was anonymisation of the participant IDs and their linkage with a particular participant. The research team was on standby and in regular touch with participants for any technical help they needed to access the platform or fill the diaries.

### 3.3. Analysis of Initial Questionnaires

Prior to commencing the study, the participants were required to fill in an initial questionnaire (Appendix A, Table A1) focusing on their subjective opinion on the impact of poor air quality, their ethnicity, education level, combined household income, distance from the main road, and their house’s physical characteristics such as the year of construction and house type, health-related questions such as the presence of any asthma patient in the household, and the type of heating. 

The analysis shows that there was diversity in ethnicity among the participants in terms of Asian, Mixed, Arabic, and African. The ethnic multiplicity brings diversity to the study regarding variations in the cooking style, window opening hours, home interior settings, and living patterns in relation to IAQ monitoring. A total of 40% Asian or Asian British (Pakistan), 20% African, and 30% other ethnic origins (Arab other Asian). Some other demographic information of the participants has also been analysed, as shown in Table 2, to bring diversity to this study. From the table, it can be observed that there was a variation in house location from the main road in terms of distance. A total of 60% of the houses in the study are within 0.1 km of the main road, and 30% of the houses are within 0.5 km of the main road. We have one flat, two semi-detached and seven terraced houses with four electric and six gas cooker users. Additionally, there were different heating systems, covering 8eight central, one electric, and one gas heating.

### 3.4. Study 1: Data Analysis and Discussions

The data have been divided into two individual months for further analysis. The final data assessment has been categorised into three steps (i) IAQ readings analysis, (ii) indoor activities analysis, and (iii) analysis of the increase in awareness.

#### 3.4.1. IAQ Readings Analysis

**Figure 2:**
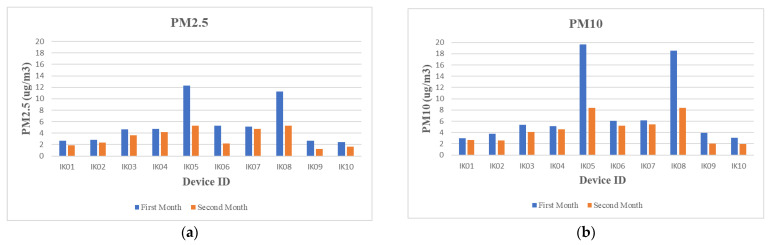
(**a**,**b**): IAQ (PM_2.5_ and PM_10_) improvement patterns from all participant households, with blue plots showing the first-month readings and the orange plots showing the second-month readings.

It can be observed from Figure 2 that there was an improvement in the indoor air quality data in the second month as compared to the first month’s data. The average indoor air pollution readings have been compared to explore if there was any significant change in indoor air pollution levels. The improvement was not uniform across all the households. The analysis shows that there was a minimal improvement of 0.4 µg/m^3^ to the maximum of 6.96 µg/m^3^ for PM_2.5_ and a minimal of 0.26 µg/m^3^ to a maximum of 11.2 µg/m^3^ for PM_10_. The indoor air pollution readings are also plotted, as shown in Figure 2a,b. From these figures, it can be observed that the average indoor pollution level (PM_2.5_ = 60% and PM_10_ = 80.43%) improved in the second month of the study compared to the first month among all the participant’s households. Additionally, a t-test was conducted, which demonstrated that the first month’s average indoor pollution level (PM_2.5_: M = 5.41, SD = 3.54, and PM_10_: M = 7.46, SD = 6.24) was higher compared to the second month’s average indoor pollution level (PM_2.5_: M = 3.26, SD = 1.57, and PM_10_: M = 4.54, SD =2.39). This analysis showed a significant improvement in IAQ, PM_2.5_: t(9) = 2.82, *p* = 0.01, and PM_10_: t(9) = 2.24, *p* = 0.026, respectively.

#### 3.4.2. Indoor Activities Analysis

From the indoor air pollution data, it is easy to observe an improvement in indoor air quality, as shown in Figure 2. We have further analysed the air quality improvement concerning how participants performed different activities using the daily digital diary in two different months. One of the key activities we analysed was the window-opening activity. The participants were asked how long they kept windows open as part of the daily digital diary. 

All participants compared the window opening hours between the first and second months. The analysis has shown that there has been an improvement in window opening hours ranging from 11% to 39%. In addition, the comparison demonstrated that there was a greater frequency of window opening in the second month compared to the first month. The statistical analysis of the first month’s average window opening hours (M = 62.1, SD = 22.52) in comparison to the second month’s average window opening hours (M = 79.6, SD = 24.94) significantly demonstrated that there was an improvement in the window opening hours, t (9) = −7.13, *p* = 0.000055 (< 0.001) in the second month.

#### 3.4.3. Measuring Awareness with Qualitative Analysis

A semi-structured interview regarding participants’ experience during the study to evaluate their awareness of indoor air quality was conducted at the end of the study. Interviews were arranged online due to the COVID-19 pandemic. Following the interview session, we analysed the feedback to explore the four categories of awareness.

(i) IAQ awareness

According to the WHO, indoor air pollution causes significant damage to human health globally. In general, when it comes to showing awareness of IAQ, understanding the seriousness of the pollutants present in the indoor air, the impact of poor air quality on health, and indoor activities that lead to poor air quality are key areas to demonstrate awareness. Considering IAQ awareness, 5 out of 10 participants have a good level of IAQ awareness, 2 have partial awareness but have a concern regarding IAQ, and the remaining 3 participants have an awareness of outdoor AQ but not indoor AQ. This initial awareness level was considered during the semi-structured interview to explore the change in awareness level.

**Figure 3:**
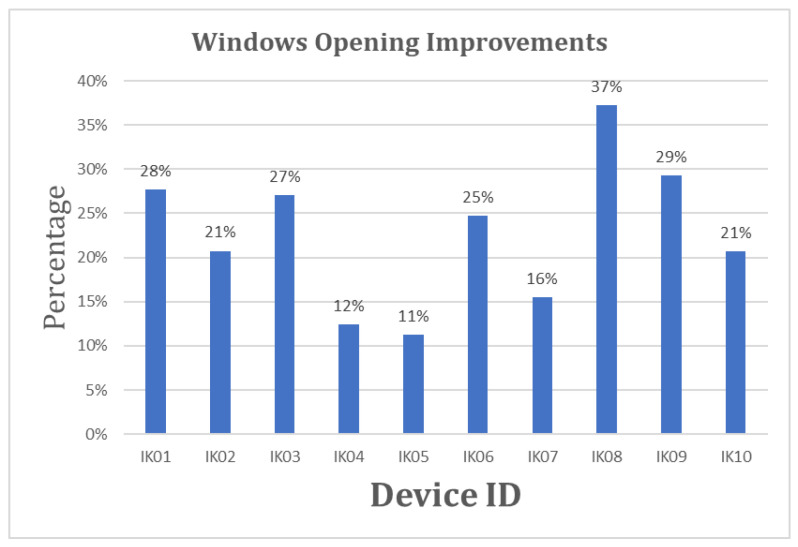
Shows improvement compared to the first month of deployment in the second month.

From the interviews, it is clear that the intervention of showing indoor air pollution data and asking them to fill in a daily digital diary of indoor activities brought further awareness, as shown in Figure 2 and Figure 3. During the interview interactions, the talk was organised to evaluate the participant’s engagement with the study, changes in their day-to-day household indoor activities, and their observation on the correlation of their indoor activities with indoor pollution levels considering the graphs and daily digital diary. Participants highlighted some interesting patterns and expressed concerns about their understanding of indoor activities. For example, there is a frequent mention of products, i.e., sources, that could cause bad air quality (*“We need to get more fresh air, we need to minimise the use of products that removes grease/grime and over that effect on health”*); linking to the data and how they are assured or concerned by the air quality (*“I really want to ensure that we have good air quality”*, *“If it is normal and not exceeding the WHO or UK threshold then I am ok with that”*, *“That does help me in terms of bringing in awareness of maintaining the air quality by comparing with WHO guideline and will able to see you are comparing with previous and current readings level”*); or being conscious about what they do in house (*“Making more aware of all the things we doing inside the house”*). These comments clearly show that the participants understand and are concerned about IAQ. This was also confirmed while collecting devices back from the participants’ houses.

(ii) Awareness through Indoor Activities: opening windows, cooking, and cleaning

Indoor activities such as window opening frequency and duration, cooking, cleaning, or vacuuming impact the IAQ. Although no specific advice was provided to link activities with IAQ, apart from asking them about these activities in their daily digital diary, there was an apparent increase in awareness in their feedback. For example, their comments reflect this increase in awareness (*“When I vacuum, I think about air quality”*; *“Prior to this, I never thought about window opening and air quality has relation”*; *“definitely…I am always in the home recently because of the pandemic. I have noticed the change reflected upon me...obviously…this is great for the houses who need it the most”*). The increase in opening windows, as demonstrated through quantitative results shown in Figure 3, is also reflected in their qualitative feedback (*“Before I cooked, I never open windows, but now I do after this”*; *“When guests come over or partying and stuff and normally the last thing in my mind is to get fresh air in and open the window”*; *“I like cleaning and I used to use bleach but Now I open windows when I use it”*). This is complemented by an increased awareness of using exhaust fans where possible (*“Now after this, we start turning the gas fan on more frequent”*). The participants have also highlighted that the visualisation also impacted their reflection (*“Since that device, it’s a conscious act for me and what I am using and what I am doing”).*

(iii) Spreading awareness

We also analysed how the participants contribute to spreading awareness through connections such as family and friends (*“It’s been discussed a lot in the family since the device deployed”*). For example, one of the participants said that if someone talks about asthma, they discuss indoor air quality and household activities. It was also a recurring theme that participants recommended that others measure the indoor pollution level and see what activities make it worse (*“More concern about the indoor air quality and found my family and friends visiting us have a concern as well”*; *“I would recommend to others that it’s a good idea to monitor your IAQ because it allows you to be conscious about what you do not see”*; *“My friend come to my house and asked about this device and I explain what is it doing and asked me how can she get such device for their house as well”*; *“I discussed this with my close friends and they asked for market availability”*).

(iv) Role of technology in raising awareness

Using the pre-study questionnaires, we measured participants’ awareness of the importance of air quality. With the deployment of IAQ devices, we observed a general curiosity about how the device works. One participant noted the following: *“I was quite comfortable with the device and get familiar once I started to fill daily digital diary”*. While another participant commented the following: *“In terms of appearance, it’s quite big and looks like a household appliance device” “The user-friendly graphical display was very well received for the information it provides, particularly in the context of standards”*. The daily activity log in the form of a digital diary helps them to log their indoor activities with any device such as an iPad, mobile, or personal computer. Many participants found that this reflective experience enabled them to research indoor air pollution as an issue and be aware of it. One participant said the following: *“I already know what air quality means so I was excited to monitor it so that I will have an idea of the quality of air inside my house”*. Similarly, another participant stated the following: *“Just concerned to know what activities trigger air pollution and how it changes on the dashboard”*. There was also informal confirmation in some of the behaviour changes, with one participant stating the following: *“We cook a lot, if I need to cut down something to make air quality better then I am happy to do that and I feel this study helps me to achieve that”, “So much behaviour change in me as a mother”*, *“I was talking with my mum regarding cooking methods”,* and *“I forgot to use the cooking fan and now I have used to use it even for two min egg fry”*

Some suggestions for improvement included focusing on the activities we cover in the daily digital diary: *“need more activities in daily activity log with more options”*. New requirements were also expressed: *“note section to use to be recorded for yourself”*. Finally, the majority of the participants preferred a mobile application instead of a web-based diary/tool: *“Mobile application is more convenient and cooler.”*

This first study focused on raising the awareness of IAQ with a digital visualisation platform and daily digital diary. Real-time IAQ were shown to the household participants on a digital visualisation platform, including the average over a week, and in the context of the WHO and UK government recommended limits. In addition, the participants also filled out a daily digital diary that was triggered by self-understanding and reflection. By analysing the study data of all the participants, we noticed that there had been an increase in awareness of IAQ in relation to their indoor household activities. However, the rise in awareness leading to behaviour changes might have been accidental. To strengthen this finding, we have conducted a second study with the help of a formal behaviour change model, COM-B. We have utilised COM-B to design digital interventions, their timing, and form formally, and measured the behaviour change. This design has also been influenced by the outcome of study 1, where the window opening hours have appeared as an influencing factor in improved IAQ. With the help of a behavioural change framework, digital intervention procedures have been designed to complement the findings of study 1.

## 4. Design of Study 2: Using COM-B Model for Behaviour Change

Distinct health and well-being programmes have employed the Behaviour Change Wheel (BCW), including the COM-B model, to enhance particular behavioural patterns in various populations. IoT-based LCS technologies have been used to monitor IAQ to bring air pollution awareness to the citizens. Having said that, the COM-B model is helpful to applied researchers and developers in at least three ways. Firstly, it explains the assumptions behind behaviour change interventions and how they relate to general human motivation ideas. Secondly, much of the theory behind the model is unique to this area of research, so the model gives a set of concepts that can be communicated to people who are unfamiliar with the field. Finally, the model provides guidance on which types of intervention will most likely be effective for specific groups or behaviours [75]. The COM-B approach is heavily utilised in public health messaging and for observing behavioural changes [76,77]. These include digital and analogue interventions for weight loss, quitting smoking, reducing the use of unnecessary antibiotics, and boosting physical activity levels [78,79]. Xu et al. [80] evaluated the connection between air pollution and travel behaviour. This study indicated that there was no noticeable relation between poor outdoor air quality and the citizens’ travelling distance.

### 4.1. Introduction to COM-B

The COM-B model of behaviour change is one of several social-cognitive models developed to establish the theoretical underpinnings for education, training, and public health interventions to change human health behaviours [81,82]. The basic premise is that the willingness to engage in individual behaviour change is determined by an individual’s level of **motivation**, moderated by beliefs about their **capability** to perform the desired behaviour successfully, coupled with the sufficient **opportunity** to perform the **behaviour**. In applied behaviour analysis, the COM-B model is a widely used tool for identifying what needs to change within an individual for a specific behaviour change intervention to be effective. 

**Figure 4:**
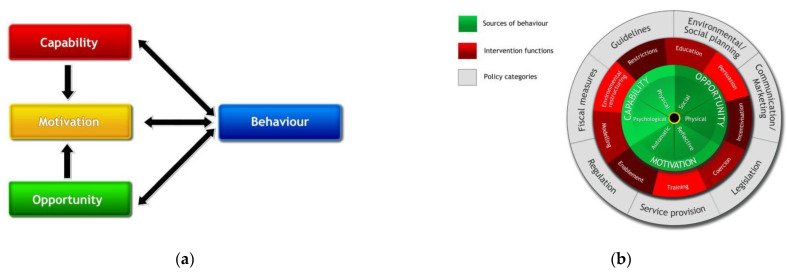
(**a**) The COM-B model [75]. (**b**) Behaviour Change Wheel (BCW) [75].

The COM-B model, along with descriptions of the entities involved and a summary of their interactions, is illustrated in Figure 4. In addition, the Behaviour Change Wheel (BCW) of the COM-B model provides an operational model for designing interventions that target COM:

**Physical Capability**: It refers to someone’s physical capability involving their physique to carry out an activity. 

**Psychological Capability**: It refers to someone’s psychological capability involving their mental functioning (understanding and memory).

**Physical Opportunity**: This focuses primarily on other physical environments—such as finance and material resources.

**Social Opportunity**: This primarily focuses on social and cultural norms involving other people. 

**Automatic Motivation**: This primarily focuses on desires and habits that organically support motivation.

**Reflective Motivation**: This primarily focuses on conscious thought processes.

The BCW consist of nine intervention functions (education, persuasion, incentivisation, coercion, training, enablement, modelling, environmental restructuring, and restrictions). These interventions and their meanings are presented in Table 3. 

### 4.2. Applying COM-B Model to Analyse the Change in Behaviour: Measuring the Change in Behaviour through Indoor Activities

We used the COM-B model and Behaviour Change Wheel (BCW) as a framework for designing digital interventions to change behaviour in two crucial aspects that improve IAQ: (1) the use of domestic products and (2) the use of ventilation. The digital interventions manifest with the help of a web portal that showcases the data coming from the IAQ monitoring sensors, which is like the first study, but they also provide more actionable information that is underpinned by the COM-B model and the BCW implementation framework.

**Figure 5:**
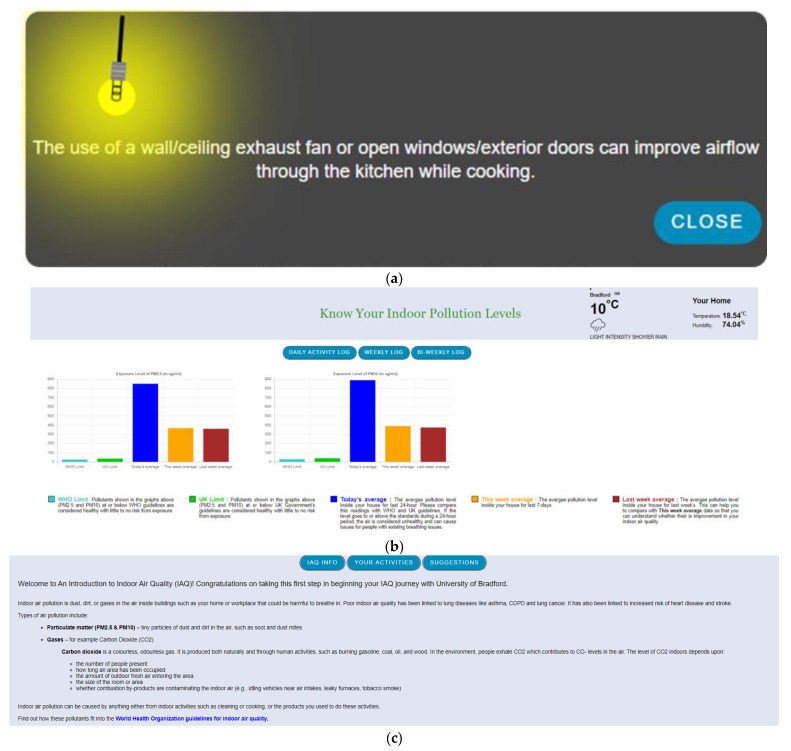

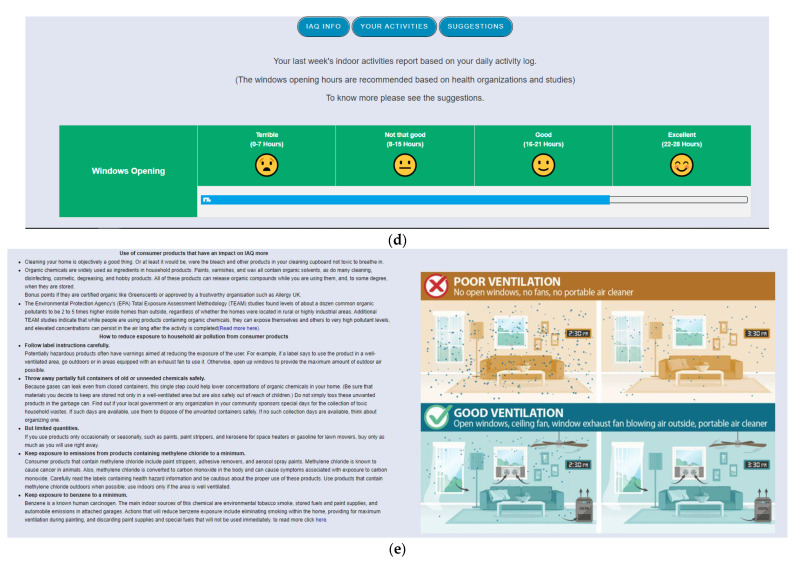
(**a**). An interactive pop-up to provide instant information about IAQ improvement. (**b**). An interactive digital visualisation platform to give access to participant’s IAQ data with relevant information to provide context to this graphical presentation. (**c**). The information regarding air pollutants helps the participant to get more knowledge. (**d**). Information about participants’ indoor activity (window opening hours) coming from the participants referring to how better they are doing in reducing indoor air pollution on the meter. (**e**). Interactive intervention with detailed information for IAQ improvement.

**Intervention 1 (Int1):** A pop-up on the screen with a new informative message to support two interventions whenever the participant logs in (see Figure 5a). Each time the participant logs in, this message will be different and will present messages supporting their psychological capability. 

**Intervention 2 (Int2):** Figure 5b shows all the IAQ information from the household. This shows the dynamics of the indoor air pollution levels with relevant information, such as the comparison of today’s average pollution data with this week’s average and the last week’s average as well. This comparison helps participants to understand how the pollution level is now compared with the previous week’s data and whether there are improvements. Additionally, comparison with the WHO and UK-guided air pollutant limit helps them understand the pollution level according to the guidelines. Finally, as shown in Figure 5c, all the information has been presented to understand the context of indoor air pollution. This is additional information provided for study 2 participants compared to study 1 participants.

**Intervention (Int3):** How well they are doing in terms of using windows to control ventilation. For example, Figure 5d shows the participants’ window opening hours in that particular week where ‘good’ performance is recorded when participants have average window opening hours between 16–21 hours. This also recommends what they need to do to move into an ‘excellent’ rating. 

**Intervention (Int4):** Suggestions—this digital intervention provides various levels of contextual information. For example, the advice shown in Figure 5e provides all the information regarding the use of ventilation to improve IAQ, and the impact of consumer products on the IAQ. 

Table 4 and Table 5 below outline how the Behaviour Change Wheel has guided the design of these four digital interventions to support behaviour change regarding ventilation and product usage in the house. 

### 4.3. Study 2 Methodology

**Figure 6:**
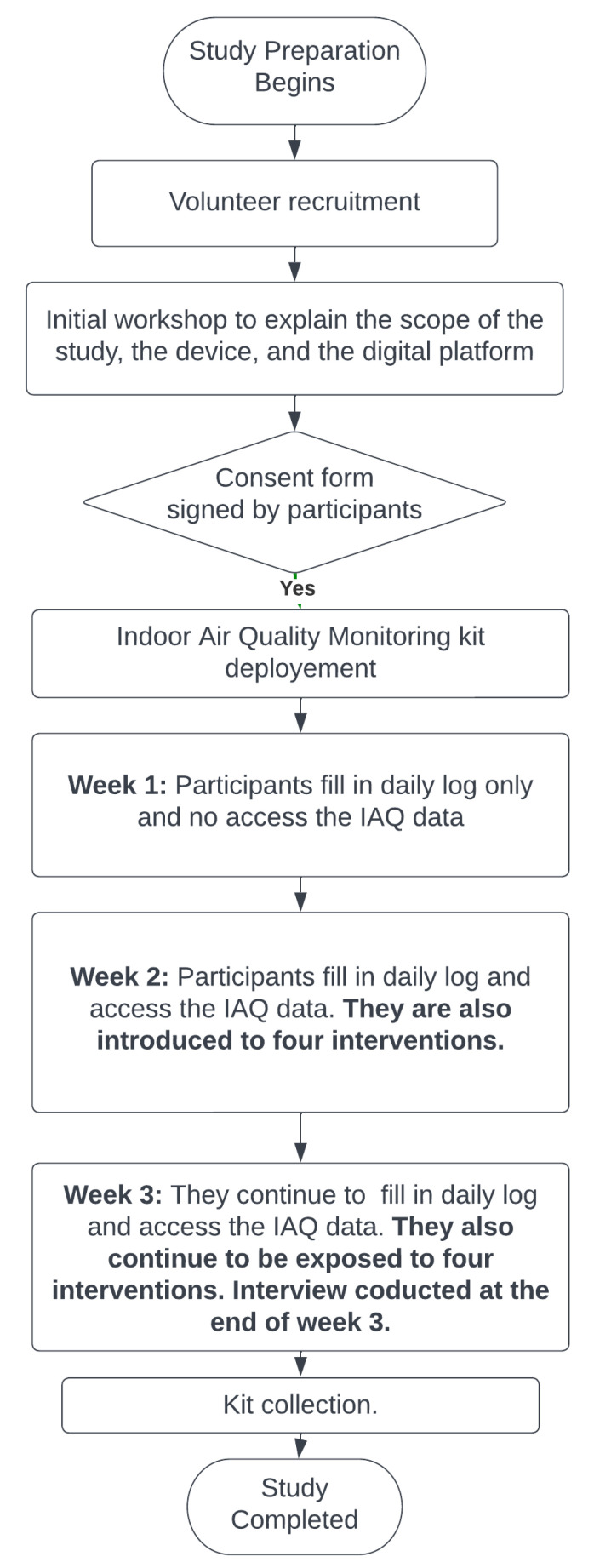
Study flow diagram for bringing intervention to raise awareness about IAQ.

This study was conducted in sequential flow, as shown in Figure 6, in May 2022 for three weeks with different participants from study 1 in the city of Bradford. This study aimed to measure behaviour changes among the citizens when supported with COM-B-based digital interventions for improving IAQ based on window opening hours (ventilation) and the reduction in the use of house products. Eight households agreed to take part in this study. Ethical approval was granted by the Chair of the Biomedical, Natural, Physical, and Health Sciences Research Ethics Panel for this study.

Online workshops were set up to partially outline the study’s specifics, including device information, a daily digital diary, and the deployment process. We have more faith in this study because of the numerous questions that the participants raised throughout the workshop concerning air quality, its importance for them, the advantages of monitoring IAQ, and other topics. Citizens are eager to participate in this study as a result of the workshop. To install the LCS-based IAQ monitoring device, 8 households were chosen based on socioeconomic and demographic factors such as their location, ethnicity, and kind of home, as shown in Table 6. None of the participants used dehumidifiers in their households to control indoor humidity. Individuals were scheduled with each participant with the permission of the citizens who agreed to participate in the study.

Following the individual sessions, the planning for the LCS-based AQ monitoring device deployment began. For this study, the same kit was used to monitor PM_2.5_ and PM_10_. The team went to the participants’ homes and deployed the devices. The participants were also asked to fill in a pre-study initial questionnaire—the same as in study 1. In addition, the research team provided instructions on how to complete the daily digital diary after the device deployment process. Other than this information, no further details were disclosed to the participants. Each participant had their login information for the digital visualisation platform, thus protecting their privacy. Additionally, any questions or concerns from participants were handled through telephone or email communication. 

During the first week of the study, participants did not have access to any IAQ data from their households, and they only completed a daily digital diary to log indoor activities. The IAQ data were only shown at the beginning of the second week with all four interventions through the visualisation platform. At the end of the third week, the team again set up an online meeting to conduct interviews with participants using pre-prepared questions at convenient times. We collected our IAQ monitoring devices from the participants’ homes by the third week’s conclusion.

### 4.4. Data Analysis and Discussion

Three weeks of IAQ data and indoor activity data have been collected from all the participants’ houses and analysed. 

#### 4.4.1. Behaviour Changes Related to the Use of Ventilation

**Figure 7:**
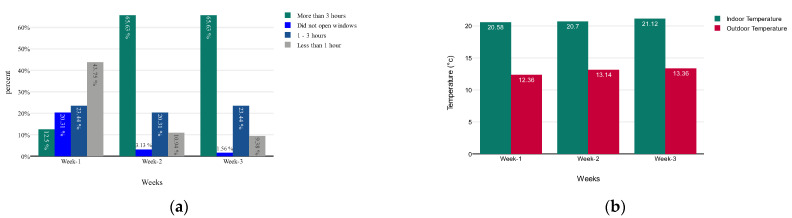
(**a**) Changing pattern of week-wise window opening hours; (**b**) comparative temperature plot between indoor and outdoor from different weeks.

As the analysis shows, from the second week, when four interventions were introduced to the participants using an interactive digital visualisation platform, there was a general increase in ventilation (opening of windows), as shown in Figure 7a. 

For the two most significant behaviour change activities, encouraging people to keep their windows open longer (more than 3 h) and discouraging them from opening their windows during the day, we have seen a statistically significant difference from week 1 to week 3. The window opening activity lasting more than three hours increased from 12.5% to 65.63%, and the not opening window activity decreased from 20.31% to 3.13%. We also analysed the average ambient and indoor temperatures during these three weeks of study. From these two temperature analyses, it has appeared that there is no significant difference in either the indoor or outdoor temperature that can influence the actions of windows opening, as shown in Figure 7b. 

We also analysed qualitative data from the interviews conducted at the end of the study. We identified the linkage between interventions contributing to the observed behaviour change from the mentions. Intervention 4 **(Int4),** which provides advisory on improving IAQ with respect to ventilation and product use, was the most frequently mentioned intervention, followed by **Int2** (shows them the dashboard of the IAQ in their house), **Int3** (how well they are doing in that particular week), and **Int1** (a pop-up with information nuggets). 

**Int 4:** There is a general acknowledgement of the actionable information provided by this intervention *(“Good, dashboard (visualisation platform) says about how to good ventilation can improve air and how to control on products which have a direct impact on our house air”). In their feedback, they refer to the advice they have received from Int4 (“To be honest, I did. We do not do exactly the same thing in our daily life as we use to do before, my wife and I clean the house together and whenever we do, we now open windows more compare to before and generally we use bleach as a cleaning product…now we are using less or whenever use it, we put the mask on and open windows”).*

**Int 2:** Dashboard was a popular form of checking the status *(“That was so interesting and good to see my house’s pollution level in graphical view …. Do you know sometimes when I was cooking I come to my mind I need to check the pollution level on the dashboard and I open it and check it and when it shows high I open the windows I open doors.”; “Generally I never asked my wife that did you turn on the exhaust while you cook but now I always asked and see the dashboard whether are impact or not…and you get surprised…the pollution was low”). This intervention has its utility maximised by information from other interventions. For example, one stated: “One day … I tried to understand and found that whenever I do the vacuuming and mum cooking, I have breathing issues and pollution was high when I checked dashboard pollution data. So I read the information again and realised that I am not opening windows. Since then, I am doing less vacuuming or if I do, I open windows even when my mum cooks”.*

**Int3:** This intervention is used as a barometer of their performance and is frequently mentioned: *“It gives you the upgrade data and it tells you what, for example, how important to open a window and how the air pollution or the humidity is inside in your house, and I think it’s beneficial and this is based on actual true record”*.

#### 4.4.2. Behavioural Change Analysis—Use of Products in the House

We analysed the answers to the post-study interview question: “Did you change any of your regular day-to-day activities after seeing data on your IAQ level?”. In the case of all households, there was mention of their behaviour change regarding the use of products that potentially impact IAQ. Table 7 shows the comments made by each of the households.

#### 4.4.3. Behavioural Change Analysis—Improvement in IAQ

**Figure 8:**
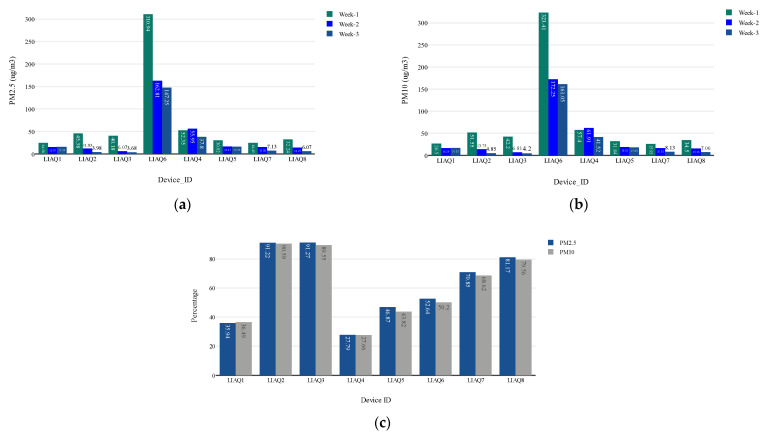
(**a**) Average PM_2.5_ data from different households in week 1, week 2, and week 3; (**b**) average PM_10_ data from different households in week 1, week 2, and week 3; (**c**) percentage change in the indoor pollution level of PM_2.5_ and PM_10_.

Behavioural change among the citizens was analysed in relation to the digital intervention reflecting the pollution readings measured using IoT-enabled LCS devices, as shown in Figure 8a,b. From these figures, it can be observed that there is a pattern of drops in the indoor air pollution levels for all pollutants (PM_10_ and PM_2.5_) across all the participants’ houses. In addition, the overall percentage drops in pollution levels from week 1 to week 3 are also analysed, as shown in Figure 8c. This figure reflects that there has been a drop of 27.79% to 91.27% for PM_2.5_ and 27.66% to 90.59% for PM_10_ among all the households. These numbers clearly demonstrate that the citizens’ awareness and digital intervention in ventilation improved noticeably when we combined the IoT system with the COM-B model. After analysing a week-wise drop in indoor air pollution levels, a drop rate from week 1 to week 2 and a further drop from week 2 to week 3 can also be observed.

In a further analysis, we analysed the potential impact of humidity on the PM values since studies [83,84,85] have shown that the PM values vary with relative humidity (RH). Furthermore, these studies have reported a reduction in the PM values with a decline in the RH value. Considering this, the PM concentrations were assessed in relation to RH, which gave a similar pattern of the reduction in the PM values with the reduction in RH, as listed in Table 8. However, there were non-uniform changes in the RH and PM values because of the different house types and indoor activities.

The literature also argues that the window opening hours are related to indoor RH [86,87,88,89]. Therefore, we also explored the impact of the window opening hours (the intervention) on the RH readings in our study to analyse whether any patterns show a relationship between the window opening hours and the RH values. After analysing the window opening hours, we found that each participant increased their window opening hours after introducing the digital interventions, as shown in Figure 7a. This change in window opening hours eventually impacts the indoor RH value. Considering these two analyses, it has been postulated that the intervention to encourage the opening of windows also reduced the RH values and hence the PM values, leading to an improved IAQ.

This weekly analysis shows that there were marginal or significant improvements in indoor air pollution across all the households, which could be a potentially false indicative improvement. To eliminate this factor, each household’s daily percentage change in indoor air pollution was computed as listed in Table 9 for PMs. From the table, it can be observed that there was a daily basis rise and fall in indoor air pollution readings across all the households. However, some household readings, such as LIAQ1 and LIAQ4, are not uniformly improved, as we observed in the weekly air quality improvement analysis. On the other hand, some households, such as LIAQ3 and LIAQ6, have a better daily reading improvement, which is also reflected during the weekly analysis. From these two tables, it can be observed that there was an improvement in the daily IAQ readings as digital interventions were introduced.

## 5. Conclusions and Future Work

Ambient air pollution is receiving widespread attention throughout the world. However, indoor air pollution, which is an essential aspect of health and well-being because we spend 90% of our average time indoors, has yet to receive the same attention. Human indoor activities, ranging from the use of products to cooking, cleaning, and ventilation, significantly impact IAQ. We present one of the early works, study 1, on using digital technologies, including IoT, to raise awareness about this critical topic. We also showcase, in study 2, for the first time, as per our literature review, how a behaviour psychology model such as COM-B can be operationalised as a means of digital interventions to support behaviour change. 

We have conducted two studies—first, to evaluate the effectiveness of the IoT-enabled LCS technology for IAQ data visualisation and the reflection capability of human beings when they fill a daily digital diary to bring about IAQ awareness. In the first study, we demonstrated an increased understanding of indoor air pollution that allowed participants to do their research, leading to behaviour changes that resulted in an improved IAQ. However, this was performed only based on an IoT-enabled digital visualisation and monitoring system without the scaffolding of a formal behaviour change model. In the second study, we used a widely used behaviour change model, the COM-B model, which also provides the BCW method to design targeted digital interventions. As a result, we have developed four digital interventions that are manifested through the digital platform. These interventions are grounded to support psychological capability and social opportunity presented by the BCW method. We have demonstrated that the COM-B model can change behaviour concerning two critical and impactful activities—domestic product usage and ventilation. The analytical results from study 1 and study 2 have shown that there has been a significant improvement in indoor air quality. In study 1, the average improvement was 60% for PM_2.5_ and 80.43% for PM_10_ in the second month compared to the first month, along with improvements in the window opening hour ranging from 11% to 39% among all participants. Similarly, in study 2, the indoor air pollution levels were reduced, ranging from 27.79% to 91.27% for PM_2.5_ and 27.66% to 90.59% for PM_10_ among all the participants’ houses. The daily percentage change in indoor air pollution readings was also analysed across all the households, which revealed no consistent improvement. Still, as the week progressed, the IAQ improved. This improvement was because of the interventions introduced before the start of week 2. From these two study results, it can be observed that the IoT-enabled IAQ monitoring system improved the indoor air quality and also raised self-awareness. Still, no systematically accomplished methodology has been implemented to motivate behavioural change. Using the COM-B model in complementing the IoT-enabled IAQ monitoring system in study 2 helps identify how digital interventions can be used as a formal method and inspire behavioural change among citizens. Using the COM-B model with digital interventions improved indoor air pollution levels more than no interventions.

In both studies, as a limitation, no control mechanism was applied to mitigate or reduce pollutant emissions. In addition, both of these studies have not considered any outdoor pollutants or sources of pollutants such as O3, NO2, PM, or vehicle emissions from road traffic. For future and ongoing work, we are working on extending the study with more activities measuring additional indoor air pollutants to target and quantify behaviour between asthmatic and non-asthmatic patients. Additionally, other factors such as socioeconomic status, house type, and indoor activities, such as cooking and cleaning, will be incorporated to improve IAQ and behavioural changes. This study will also be further enhanced with the diverse participation of citizens over a long period.

## Figures and Tables

**Table 1:** List of low-cost sensors and their details used to conduct these studies.

Sensor Name	Description	Sensor Specification	Image
BME680	This sensor can measure temperature, humidity, barometric pressure, and VOC gas.	Temperature in Celsius (*C), Humidity %, and Barometric pressure hPa	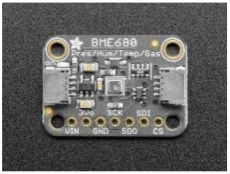
SDS011	This sensor is used to measure PM_2.5_ and PM_10_ air pollutants. This sensor is an infrared-based laser sensor and has a fan to provide self-airflow.	PM_2.5_: μg/m^3^PM_10_: μg/m^3^	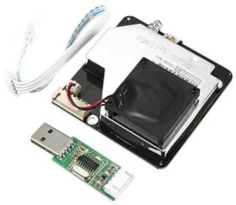

**Table 2:** Summary of initial questionnaires outcome of participant’s demographic information for study 1.

Sensor ID	House Location	Type of House	Type of Cooker	Type of Heating
IK01	Within 0.1 km from the main road.	Terraced	Electric	Central heating
IK02	Within 0.1 km from the main road.	Terraced	Gas	Central heating
IK03	Within 0.1 km from the main road.	Terraced	Gas	Central heating
IK04	Within 0.1 km from the main road.	Semi-detached	Gas	Central heating
IK05	Within 0.1 km from the main road.	Flat	Electric	Electric heating
IK06	More than 0.5 km from the main road	Terraced	Gas	Central heating
IK07	Within 0.1 km–0.5 km from the main road.	Terraced	Gas	Central heating
IK08	Within 0.1 km–0.5 km from the main road.	Terraced	Electric	Central heating
IK09	Within 0.1 km–0.5 km from the main road.	Semi-detached	Electric	Central heating
IK10	Within 0.1 km from the main road.	Terraced	Gas	Gas Heating

**Table 3:** The nine different types of intervention included in the BCW.

	Intervention Functions
Education	Increase knowledge or understanding
Persuasion	Using communication to induce positive or negative feelings to stimulate action
Incentivisation	Creating an expectation of reward
Coercion	Creating an expectation of punishment or cost
Restriction	Using rules to reduce the opportunity to engage in the behaviour (or to increase behaviour by reducing the opportunity to engage in the competing behaviours
Environmental restructuring	Changing the physical or social context
Modelling	Provide an example for people to aspire to or emulate
Enablement	Increasing means or reducing barriers to increase capability (beyond education or training) or opportunity (beyond environmental restructuring)
Education	Increase knowledge or understanding

**Table 4:** COM-B model elements help design interventions for the domestic products that citizens use indoors.

Intervention Functions				
	Education	Persuasion	Modelling	Enablement
**Psychological** **Capability**	**Int 1 and Int4** (These increase knowledge and understanding about the impact of product usage on indoor air quality)	**Int2 and Int3** (These provide relevant and contextualised information about product use and impact on air quality to simulate action)	-	**Int2 and Int3** (The information increase capacity to change by providing important insights)
**Social** **Opportunity**	**Int 1 and Int4** (These increase knowledge and understanding about the negative impact of product use that allows them to share with their social group)	**Int2 and Int3** (These provide relevant and contextualised information on how the product usage change brought positive results, to showcase their social group for simulating actions)	**Int2 and Int3** (If they have seen a positive change in IAQ in their own home, then they act as an exemplar to their social group)	-

**Table 5:** COM-B model elements help to design interventions for indoor ventilation, which can help to reduce IAQ.

Intervention Functions				
	Education	Persuasion	Modelling	Enablement
**Psychological** **Capability**	**Int 1 and Int4** (These increase knowledge and understanding about the impact of mechanical and natural ventilation on indoor air quality)	**Int2 and Int3** (These provide relevant and contextualised information about ventilation use and impact on air quality to simulate action)	-	**Int2 and Int3** (The information increase capacity to change by providing important insights)
**Social** **Opportunity**	**Int 1 and Int4** (These increase knowledge and understanding about the negative impact of the lack of ventilation that allows them to share with their social group)	**Int2 and Int3** (These provide relevant and contextualised information on how the increase in windows opening during certain activities brought positive results, to showcase their social group for simulating actions)	**Int2 and Int3** (If they have seen a positive change in IAQ in their own home, then they act as an exemplar to their social group)	-

**Table 6:** Summary of initial questionnaires outcome of participant’s demographic information for study 2.

Sensor ID	House Location	Type of House	Type of Cooker	Type of Heating
LIAQ1	Within 0.1 km from the main road.	Terraced	Gas and Electric	Central heating
LIAQ2	More than 0.5 km from the main road	Terraced	Electric	both central and electric
LIAQ3	Within 0.1 km–0.5 km from the main road.	back-to-back house	Gas	Central heating
LIAQ4	Within 0.1 km from the main road.	Semi-detached	Gas	both central and electric
LIAQ5	Within 0.1 km from the main road.	Semi-detached	Electric	Central heating
LIAQ6	Within 0.1 km from the main road.	Semi-detached	Gas	Gas Heating
LIAQ7	Within 0.1 km from the main road.	Semi-detached	Gas	Central heating
LIAQ8	Within 0.1 km from the main road.	Detached	Gas	Central heating

**Table 7:** Answers to interview questions: “Did you change any of your regular day-to-day activities after seeing data on your IAQ level?

Household/Device ID	Comments
LIAQ1	*“Even now I am checking the content of the cleaning products”* *“Yes, we are using less bleach or cleaner in the kitchen for cleaning”*
LIAQ2	*“Windows opening more and less use of candles in my house”*
LIAQ3	*“use water for cleaning unless cleaner is required”* *“less oil usage”*
LIAQ4	*“My wife and I clean the house together and whenever we do, we now open windows more compare to before and generally we use bleach as a cleaning product…now we are using less or whenever use it, we put the mask on and open windows”*
LIAQ5	*“Yes, I, as a mother and housewife, I love my house to smell nice all the time so I used a candle or Incense Sticks. Since I noticed that this raises the pollution high, I am using very less”* *“Even I practically checked with the dashboard, whenever I burn them, the pollution level looks high on the graph”*
LIAQ6	*“My wife loves cleaning, she always the tidy kitchen and keeps it clean. After I show what is written on the dashboard, she is now more concerned about using cleaning products”*
LIAQ7	*“Use less cleaning products or whenever use put gloves and mask on.”*
LIAQ8	*“Me and my family are now more concern about bleach use”*

**Table 8:** The weekly average reading of RH, PM_2.5_, and PM_10_ from IAQ devices.

Device ID	RH			PM_2.5_			PM_10_		
	Week 1	Week 2	Week 3	Week 1	Week 2	Week 3	Week 1	Week 2	Week 3
LIAQ1	60.64	53.28	52.97	24.58	15.07	15.54	26.5	16.29	16.83
LIAQ2	67.65	57.81	58.01	45.38	11.83	3.98	51.55	13.75	4.85
LIAQ3	64.31	55.79	55.42	40.18	6.07	3.68	42.2	6.81	4.2
LIAQ4	61.82	63.29	55.27	52.35	55.95	37.8	57.4	61.91	41.52
LIAQ5	57.14	54.56	52.27	30.02	16.47	15.95	31.84	18.81	17.89
LIAQ6	74.95	69.14	68.65	310.94	162.81	147.25	323.41	172.25	161.05
LIAQ7	66.04	64.73	55.5	24.45	15.08	7.13	25.92	16.35	8.13
LIAQ8	57.93	54.16	52.33	32.24	14.25	6.07	34.5	15.36	7.06

**Table 9:** Daily percentage change in PM_2.5_ and PM_10_ readings from all households.

	Device ID															
	LIAQ1		LIAQ2		LIAQ3		LIAQ4		LIAQ5		LIAQ6		LIAQ7‘		LIAQ8	
Pollutants	PM2.5	PM10	PM2.5	PM10	PM2.5	PM10	PM2.5	PM10	PM2.5	PM10	PM2.5	PM10	PM2.5	PM10	PM2.5	PM10
1	▼ 86.20%	▼83.97%	▲ 98.30%	▲ 95.39%	▲ 19.66%	▲ 28.22%	▲ 220.20%	▲ 240.77%	▼ 24.62%	▼ 25.80%	▲ 28.22%	▲ 220.20%	▼ 49.28%	▼ 44.85%	▲ 217.16%	▲ 217.12%
2	▲ 769.05%	▲674.95%	▼ 46.49%	▼ 50.56%	▼ 35.43%	▼ 40.39%	▲ 28.74%	▲ 30.48%	▼ 11.84%	▼ 13.75%	▼ 40.39%	▲ 28.74%	▼ 10.40%	▼ 14.16%	▼ 35.20%	▼ 38.28%
3	▼ 29.68%	▼32.13%	▲ 177.14%	▲ 209.04%	▲ 26.53%	▲ 28.96%	▼ 61.08%	▼ 61.96%	▲ 3.04%	▲ 2.35%	▲ 28.96%	▼ 61.08%	▲ 48.57%	▲ 49.85%	▼ 12.98%	▼ 16.82%
4	▲ 76.55%	▲79.43%	▼ 71.52%	▼ 74.36%	▼ 36.09%	▼ 36.43%	▲ 67.35%	▲ 70.39%	▲ 73.79%	▲ 69.61%	▼ 36.43%	▲ 67.35%	▼ 41.28%	▼ 40.19%	▼ 24.36%	▼ 18.68%
5	▲ 4.38%	▲8.01%	▼ 91.90%	▼ 83.19%	▲ 95.88%	▲ 94.41%	▼ 38.72%	▼ 42.41%	▼ 49.37%	▼ 46.49%	▲ 94.41%	▼ 38.72%	▼ 71.66%	▼ 68.67%	▼ 43.99%	▼ 40.15%
6	▼ 58.40%	▼56.71%	▲ 3769.80%	▲ 1913.52%	▼ 47.21%	▼ 43.58%	▼ 2.62%	▲ 1.14%	▲ 75.02%	▲ 68.13%	▼ 43.58%	▼ 2.62%	▲ 164.74%	▲ 131.27%	▲ 204.28%	▲ 169.71%
7	▲ 77.92%	▲83.09%	▼ 72.08%	▼ 74.83%	▼ 20.04%	▼ 21.52%	▲ 24.26%	▲ 23.19%	▼ 55.49%	▼ 53.06%	▼ 21.52%	▲ 24.26%	▼ 73.33%	▼ 71.33%	▼ 46.66%	▼ 44.32%
8	▼ 60.35%	▼62.72%	▼ 87.84%	▼ 85.12%	▼ 76.97%	▼ 75.45%	▲ 64.05%	▲ 66.16%	▲ 2.85%	▲ 2.23%	▼ 75.45%	▲ 64.05%	▲ 676.67%	▲ 623.02%	▲ 23.00%	▲ 18.69%
9	▲ 35.78%	▲36.26%	▲ 1743.59%	▲ 1478.48%	▼ 27.27%	▼ 23.75%	▼ 34.59%	▼ 35.40%	▲ 85.77%	▲ 97.10%	▼ 23.75%	▼ 34.59%	▼ 85.96%	▼ 83.68%	▲ 84.66%	▲ 93.26%
10	▼ 38.08%	▼37.57%	▼ 95.12%	▼ 94.55%	▼ 46.30%	▼ 40.00%	▲ 21.17%	▲ 26.64%	▼ 67.81%	▼ 65.57%	▼ 40.00%	▲ 21.17%	▲ 244.22%	▲ 196.85%	▼ 78.78%	▼ 79.18%
11	▲ 167.87%	▲151.40%	▲ 28.81%	▲ 46.15%	▲ 55.64%	▲ 35.48%	▼ 42.70%	▼ 46.62%	▼ 15.46%	▼ 16.93%	▲ 35.48%	▼ 42.70%	▲ 49.33%	▲ 52.13%	▼ 71.34%	▼ 63.10%
12	▼ 63.80%	▼60.87%	▼ 8.44%	▲ 25.67%	▲ 103.59%	▲ 96.64%	▲ 47.27%	▲ 53.98%	▲ 55.31%	▲ 40.76%	▲ 96.64%	▲ 47.27%	▼ 25.38%	▼ 23.40%	▲ 107.88%	▲ 61.69%
13	▼ 15.08%	▼15.33%	▲ 734.10%	▲ 386.81%	▼ 36.90%	▼ 35.96%	▼ 1.91%	▼ 0.60%	▲ 11.64%	▲ 16.46%	▼ 35.96%	▼ 1.91%	▼ 63.40%	▼ 62.12%	▼ 56.74%	▼ 56.59%
14	▲ 82.56%	▲80.75%	▼ 91.51%	▼ 85.93%	▲ 222.77%	▲ 191.65%	▲ 109.07%	▲ 113.04%	▲ 6.25%	▲ 8.86%	▲ 191.65%	▲ 109.07%	▼ 38.19%	▼ 34.63%	▲ 156.80%	▲ 160.64%
15	▲ 102.62%	▲96.03%	▲ 1.53%	▲ 1.30%	▼ 37.50%	▼ 32.28%	▼ 82.21%	▼ 82.76%	▼ 20.36%	▼ 29.28%	▼ 32.28%	▼ 82.21%	▲ 165.23%	▲ 147.90%	▼ 81.53%	▼ 76.76%
16	▼ 38.42%	▼39.02%	▲ 17.83%	▼ 14.53%	▼ 82.97%	▼ 79.22%	▲ 174.06%	▲ 178.07%	▲ 25.26%	▲ 22.39%	▼ 79.22%	▲ 174.06%	▼ 54.89%	▼ 52.68%	▲ 597.59%	▲ 446.14%
17	▼ 74.36%	▼62.65%	▲ 108.48%	▲ 80.08%	▲ 78.63%	▲ 55.58%	▼ 19.61%	▼ 20.28%	▼ 12.46%	▲ 14.59%	▲ 55.58%	▼ 19.61%	▲ 109.06%	▲ 128.18%	▲ 8.79%	▲ 13.22%
18	▲ 280.53%	▲166.86%	▼ 13.37%	▼ 15.10%	▲ 6.34%	▲ 4.91%	▼ 45.13%	▼ 49.63%	▼ 75.92%	▼ 76.77%	▲ 4.91%	▼ 45.13%	▼ 18.15%	▼ 29.46%	▼ 74.66%	▼ 74.48%
19	▼ 31.80%	▼29.84%	▼ 39.49%	▼ 35.77%	▲ 40.39%	▲ 38.89%	▲ 180.03%	▲ 209.85%	▲ 44.13%	▲ 39.18%	▲ 38.89%	▲ 180.03%	▼ 33.34%	▼ 26.64%	▲ 190.27%	▲ 185.21%
20	▼ 41.69%	▼44.60%	▲ 85.09%	▲ 130.09%	▲ 23.79%	▲ 14.97%	▼ 77.99%	▼ 79.79%	▲ 1361.36%	▲ 1163.39%	▲ 14.97%	▼ 77.99%	▲ 37.64%	▲ 30.02%	▼ 58.36%	▼ 46.12%
21	▲ 85.23%	▲88.66%	▼ 27.07%	▼ 44.57%	▲ 113.32%	▲ 108.68%	▲ 59.25%	▲ 67.50%	▼ 91.92%	▼ 91.47%	▲ 108.68%	▲ 59.25%	▼ 58.66%	▼ 57.17%	▲ 92.85%	▲ 64.08%

## Data Availability

Not applicable.

## Appendix A

**Table A1:** Initial Questionnaire.

Email address: __________	
Sensor ID: ___________	
1. Is poor air quality a concern for you? If so, why?	
_______________________________________	
2. What is the ethnicity of the household?	
o	White—English, Welsh, Scottish, Northern Irish, or British
o	White—Irish
o	White—Gypsy or Irish Traveller
o	White—Any other white background
o	White and Black Caribbean
o	White and Black African
o	White and Asian
o	Any other Mixed or multiple ethnic background
o	Asian or Asian British (Indian)
o	Asian or Asian British (Pakistan)
o	Asian or Asian British (Bangladeshi)
o	Asian or Asian British (Chinese)
o	Asian or Asian British (any other Asian background)
o	African
o	Caribbean
o	Any other Black, African, or Caribbean background
o	Arab
o	Any other ethnic group
3. What is the highest education level of the household?	
o	Entry Level
o	GCSE
o	A level
o	HND
o	Bachelor’s Degree
o	Master’s Degree
o	Doctoral Degree
4. Are there any children in the house?	
o	Yes
o	No
5. Are there any persons aged over 60 living in the house?	
o	Yes
o	No
6. What is the combined annual income of the household?	
o	Less than GPB 19,200
o	More than GPB 19,200
o	Prefer not to say
7. Where is your house located?	
o	Within 0.1 km from the main road
o	Within 0.1 km–0.5 km from the main road
o	More than 0.5 km from the main road
o	Other: _________
8. How old is the building you are living in?	
o	Built before 1955
o	Built between 1955 to 1985
o	Built after 1985
o	I do not know
9. What type correctly describes your house?	
o	Terraced
o	Semi-detached
o	Detached
o	Flat
o	Other: ______________
10. Does anyone in your family suffer from Asthma or COPD?	
o	Yes (Asthma)
o	Yes (COPD)
o	Both (Asthma and COPD)
o	No
o	Prefer not to say
11. Are there any smokers in the house, if yes, then how often do they smoke (select frequency of smoking from below)?	
o	Yes (2–3 times a day)
o	Yes (More than three times a day)
o	No
12. Do you have a (wood/log stove/burner)?	
o	Yes
o	No
13. If you answered yes to the previous question, how frequently do you refill your wood/log burner/stove?	
o	Once during single usage
o	More than once during single usage
o	Other: _____________
14. What type of cooker do you use?	
o	Gas
o	Electric
o	Both
15. What type of heating system do you have in your house?	
o	Central heating
o	Electric heating
o	Wood/log burning
o	Gas heating
o	Other: _________________
16. Do you have any pets?	
o	Yes
o	No
